# Monitoring a Railway Bridge with Distributed Fiber Optic Sensing Using Specially Installed Fibers

**DOI:** 10.3390/s25010098

**Published:** 2024-12-27

**Authors:** Kinzo Kishida, Thein Lin Aung, Ruiyuan Lin

**Affiliations:** 1 Neubrex Co., Ltd., Sakaemachidori 1-1-24, Chuo-ku, Kobe 650-0023, Japan; 2 OlitGlobal Technologies, 3Fl 329 Huaxia Road, Kaohsiung 813016, Taiwan

**Keywords:** DFOS, DAS, SHM, railway monitoring, bridge monitoring

## Abstract

This article explores the use of distributed fiber optic sensing (DFOS) technology in monitoring civil infrastructure, with a concrete example of an elevated railway bridge in Taiwan. The field test utilized multiple strain-sensing fibers attached to a 1 km span of a bullet train railway bridge, which were combined to calculate the 3-dimensional bridge deformation. The installed sensing system and continuous measurements enabled quick safety confirmation after earthquakes of Richter scale 6.4 and 6.8 magnitudes occurred. Finally, the dynamic monitoring of a bullet train using Distributed Acoustic Sensing (DAS) demonstrated the merits of fiber optic sensing for both static and dynamic measurements. The empirical data gathered through this work aid in the evaluation of DFOS technology for structural-monitoring applications.

## 1. Introduction

The design, construction, and management of civil structures nowadays rely on numerical simulations, model experiments, and personal/institutional experience to predict the lifetime, inspection, and repair schedules. However, recent achievements in distributed fiber optic sensing (DFOS), high-speed communication networks, and computing power enable near real-time, long distance, and high spatial resolution measurements [1]. It means that structures such as bridges, railways, buildings, pipelines, etc., can be instrumented with optical fibers which function as the ‘artificial nerves’ of the structure to sense strain, temperature, and vibration. The sensing data, combined with advanced data processing, allow the fast interrogation of actual structural states such as deformation and stress distribution. The frequent occurrence of natural disasters also calls for the rapid assessment of structural integrity, which can be performed by comparing normal and post-disaster measurements. In addition, long-term data can be utilized to optimize the construction of new structures, which results in more resiliency than one-ended design alone.

The structural monitoring and assessment of civil structures has traditionally used strain gauges, accelerometers, inclinometers, and cameras. Since these are point sensors by nature, scaling the monitoring system over a vast area (e.g., a kilometers-long bridge) is not trivial due to instrumentation and data assimilation difficulties. Moreover, leveraging digital twin technologies requires a lot of data points to constrain inherent uncertainties in the models [2]. On the other hand, railway bridges need high-priority SHM implementations because they are one of the critical infrastructures under continuous utilization, which makes downtime due to faults or inspections particularly costly. For instance, more than 35% of railway bridges across Europe are over 100 years old [3], which highlights the importance of monitoring and early warning systems. DFOS is an attractive solution for this problem and, hence, it is being pursued by researchers and the industry alike [4,5,6,7,8,9].

Recent studies have demonstrated the effectiveness of DFOS in monitoring several types of bridge structures [10]. Some examples include monitoring a masonry arch rail bridge in Italy under train-induced moving loads [11], vibration and temperature influence measurements of a steel-concrete composite highway bridge in Austria [12], and the loading analysis of composite bridge girders with a fiber optic sensing textile in the U.S. [13]. Optical fibers were installed inside the concrete box-girder beams of Sarajevo Bridge in Barcelona, Spain to monitor the strain evolution during the construction of a deck enlargement [14]. An example of a DFOS system integrated into a real-world bridge structure during the construction time is the case study of Nine Wells Bridge in Cambridge, UK [15]. There, multiple cables consisting of strain-sensing and loose fibers were embedded into prestressed concrete beams, and the collected data were used to analyze short-term bending effects and long-term creep and shrinkage effects under field conditions. The studies showed good signal fidelity of static and dynamic strains especially when robust fiber optic cables were used which achieved good bonding with the host structures.

A facet of DFOS technology especially suited for the distributed sensing of vibrations or acoustic emissions is Distributed Acoustic Sensing (DAS). DAS enables the interrogation of high-frequency perturbations along the entire fiber length, making it a valuable tool to extract frequency spectrum information. It has been used in a railway context for train tracking [16], broken-rail detection [17], railroad-condition monitoring [18], and detecting intrusion by animals or rockfalls [19]. As the sensitivity of DAS instruments has improved, DFOS can now detect both slow- and fast-changing signals in a high dynamic range with a single installation of a sensing cable (which may be embedded with multiple fibers) on railway structures [20].

This paper reports the static and dynamic measurement data from a DFOS field application in monitoring a 1 km section of a bullet train railway bridge structure in Taiwan. Multiple sensing fibers were installed in a way that could detect both bending and tensile strains on the bridge. The deformation of the bridge was calculated using the beam theory with the mechanical properties of the bridge cross-section. In addition, data after the occurrence of 6.4 and 6.8 Richter scale earthquakes are also reported as a case for quick safety confirmation after a natural disaster. Finally, DAS measurements of the bridge during the passing of a high-speed train showed that individual train cars can be recognized from the signals. The field data continuously collected over a month showed that distributed fiber optic sensing is crucial in order to realize long-term civil-structure health monitoring in a robust and cost-effective manner.

## 2. Materials and Methods

### 2.1. Principles of Distributed Fiber Optic Sensing

Fiber optic sensing can be broadly divided into multi-point measurement methods and distributed measurement methods [5]. The multi-point method uses Fiber Bragg Gratings (FBGs) which are lattice structures that reflect a specific wavelength at a specific point on the fiber. FBGs are a well-known method for the replacement of multiple conventional electrical sensors with an optical fiber, but the number of sensing points and fiber length cannot be very high since each grating must be tuned to a particular wavelength of light. On the other hand, distributed fiber optic sensing is a technology that utilizes the phenomenon of the reflected light (backscattered light) that partially returns when light is incident on an optical fiber. The backscattering spectrum of the reflected light changes due to strain, temperature, and acoustic events. In this report, we focus on distributed fiber optic sensing.

DFOS utilizes three types of backscattering in optical fibers: Raman, Brillouin, and Rayleigh. Raman scattering is a technique used for absolute temperature measurement that correlates the change in light intensity with changes in temperature. Brillouin scattering and Rayleigh scattering take advantage of the shift in the frequency of backscattered light due to changes in strain and temperature. Rayleigh scattering is the strongest of the three types and achieves a strain-measurement accuracy of less than 1 με, which is equivalent to or better than conventional electric strain gauges and has a localization accuracy of 2 cm [21]. This means that by installing and measuring a single optical fiber cable several kilometers long, it is possible to achieve the same measurement as if a strain gauge with a length of 2 cm is continuously arranged (250,000 points in the case of 5 km) over the entire length.

Conventional electrical sensors (strain gauges, thermocouples, etc.) have a low unit price for sensors and measuring instruments, but (1) a single sensor handles one measurement point, (2) deformation prediction is required for the selection of installation locations, (3) a large number of sensors are required for large structures, (4) electrical insulation is reduced in the long term, and (5) there are issues such as the need for explosion-proof measures. Hence, the total cost tends to increase as the measurement scale increases.

In distributed optical fiber sensing, (1) the size and location of deformation can be determined by a single optical fiber, (2) the installation is simple, (3) the cost advantage tends to occur on a large scale, (4) it has long-term durability, and (5) it is completely explosion-proof (the raw material of optical fibers is quartz glass). It is considered to have advantages in terms of performance and economy for medium- to large-scale projects [1,10].

### 2.2. Installation of Sensing Fibers on the Railway Bridge

**Figure 1:**
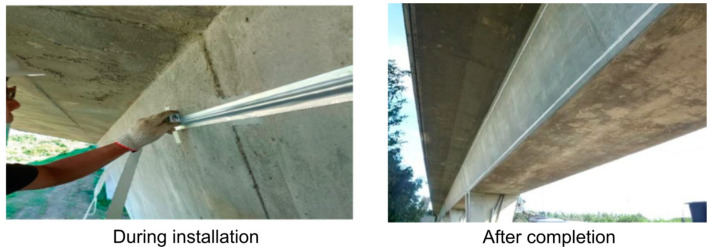
Installation of optical fiber cables on the bridge girder.

This report presents the application of DFOS to monitor the railway bridge structure of a bullet train line in southwest Taiwan. The sensing fibers were installed on a 1 km long section of the elevated railway bridge as shown in Figure 1, and the health monitoring of the structure was conducted on a trial basis over a year-long period. Neubrescope NBX-6065 and NBX-S4100 interrogators from Neubrex (Japan) were used for PPP-BOTDA (quasi-static) and DAS (dynamic) measurements, respectively. The specific acquisition parameters of PPP-BOTDA and DAS are shown in Table 1 and Table 2, respectively. The ‘spatial resolution’ refers to the gauge length (analogous to a strain gauge sensing length) over which the strain changes were measured for each sampling point. On the other hand, the ‘sampling interval’ refers to the output spatial interval between consecutive sampling points. For static PPP-BOTDA measurements, the nominal strain accuracy was in the order of tens of microstrains, and the acquisition time was about 2 min.

The fiber measures the strain along the tangential direction of the fiber at each sampling point, spaced by the designated sampling interval. In order to determine the bending curvature of the bridge in addition to the axial strain, four fiber lines were attached to four quadrants of the bridge girders. Integrating four fiber strains (detailed in Section 2.4) results in axial, horizontal-bending, and vertical-bending displacements, from which the 3-dimensional deformation of the structure can be obtained. There was a place in the monitored section where the ground was relatively poor, and it was hence designated as a priority monitoring section.

The installation of the sensing fiber was conducted as follows: smoothing the concrete surface of the structure, cleaning the surface, laying out the installation position, pretension of the optical fiber cable, fixing the fiber cable with adhesive, and covering the fiber cable with protective tape. Since the connection between the adjacent bridge girders was expected to have a large expansion or contraction and rotation, an expansion joint made of a steel plate with a thickness of 1.5 mm was prepared. The main function of the expansion joint was to provide a known relation between the gap displacements and optical fiber strains, effectively monitoring both beam deformations and their relative motion with the same fiber.

### 2.3. Temperature Compensation

The data measured by BOTDA are the Brillouin center frequencies (about 10.7~11 GHz range) in the optical fiber. Therefore, when the sensing fiber is subjected to temperature or external strain, the center frequency will change as:

(1) $$\nu =\nu_{0}+C_{\varepsilon }\times \varepsilon +C_{T}\times T,$$

where ν is the center frequency of Brillouin as measured by the instrument, $\nu_{0}$ is the center frequency at absolute zero temperature and a strain-free state, and $C_{\varepsilon }$ and $C_{T}$ are the strain and temperature coefficients of the sensing fiber, respectively. The coefficients of the fiber used in this test were $C_{\varepsilon }$ = 0.0497 MHz/µε and $C_{T}$ = 1.07 MHz/°C. By taking an initial measurement as a reference value and taking the difference between each measurement and the reference, Equation (1) can be converted to:

(2) $$\Delta \nu =C_{\varepsilon }\times \Delta \varepsilon +C_{T}\times \Delta T,$$

then the required strain difference is:

(3) $$\Delta \varepsilon =(\Delta \nu -C_{T}\times \Delta T)/C_{\varepsilon }.$$

Equation (3) gives the temperature-compensated strain change provided that ΔT is known. In this test, a segment of strain-free fiber (detached from the bridge beam) was used to acquire the temperature offset. At the same time, absolute temperature readings from an independent weather station located about 3.8 km away from the project site were also used as reference in the temperature compensation analysis. However, this method cannot account for temperature variations throughout the bridge length. Although omitted from this trial, a dedicated distributed temperature-monitoring fiber (for instance, a loose fiber in metal tube) should be employed for more accurate temperature measurements.

### 2.4. Analytical Model for Bridge Deformation

**Figure 2:**
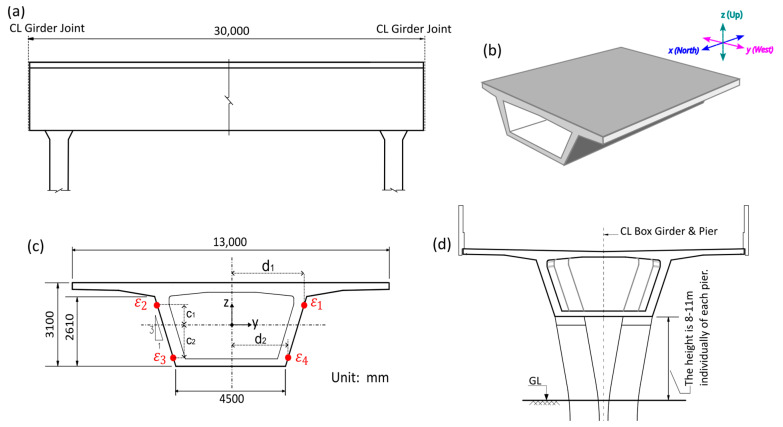
Box girder (**a**) side view, (**b**) isometric view and coordinate system, (**c**) cross-section view and designation of fiber strains, (**d**) with pier.

The optical fibers measure strain along the tangential direction of the fiber at each sampling point. By using multiple fibers, complex strain distribution can be reconstructed from fiber position in a cost-effective manner. The box girder of the bridge had a constant cross-section as shown in Figure 2 and hence can be regarded as a simply supported beam between the two pillars. Four fibers were installed in each quadrant of the box girder, to capture horizontal- and vertical-bending strains. From the four fiber strains, three unknown displacements $\left(u_{x},\;u_{y},\;u_{z}\right)$ can be calculated at each section. The torsion mode of the girder $\left(\tau_{yz}\right)$ can theoretically be obtained but is omitted in this study since the box girder has high torsional rigidity.

The analytical model to calculate the displacements of the bridge in three directions was derived from the classical beam theory. The designation of each fiber strain $\varepsilon_{i},\;i\in \{1,2,3,4\}$ is illustrated in Figure 2c. For axial displacement (lengthwise along the bridge), the fiber direction is the same as displacement direction. The four fiber strains were averaged to remove the bending component and integrated once to calculate axial displacement, as shown in Equations (1) and (4):

(4) $$u_{x}=\int \varepsilon_{axial}\;dx=\int \frac{1}{4}(\varepsilon_{1}+\varepsilon_{2}+\varepsilon_{3}+\varepsilon_{4})\;dx.$$

The curvature–strain relationship indicates that horizontal curvature $\kappa_{h}$ and vertical curvature $\kappa_{v}$ can be calculated from the separation distance of corresponding fiber pairs. Since there are two pairs of fibers for each curvature, four curvatures can be obtained:

(5) $$\kappa_{h}^{top}=\frac{\varepsilon_{1}-\varepsilon_{2}}{2d_{1}},\;\kappa_{h}^{bottom}=\frac{\varepsilon_{4}-\varepsilon_{3}}{2d_{2}},$$

$$\kappa_{v}^{left}=\frac{\varepsilon_{3}-\varepsilon_{2}}{c_{1}+c_{2}},\;\kappa_{v}^{right}=\frac{\varepsilon_{4}-\varepsilon_{1}}{c_{1}+c_{2}}.$$

From Equation (5), average curvatures are taken and integrated twice to obtain horizontal and vertical displacements:

(6) $$\kappa_{h}=\frac{1}{2}\left(\frac{\varepsilon_{1}-\varepsilon_{2}}{2d_{1}}+\frac{\varepsilon_{4}-\varepsilon_{3}}{2d_{2}}\right),\;\kappa_{v}=\frac{1}{2}\left(\frac{\varepsilon_{3}+\varepsilon_{4}-\varepsilon_{2}-\varepsilon_{1}}{c_{1}+c_{2}}\right),\;\;u_{y}=\iint \kappa_{h}dx,\;u_{z}=\iint \kappa_{v}dx.$$

The numerical evaluation of Equation (6) needs adjustment for correct boundary conditions. Since each girder is supported by two piers and connections between girders are not rigid, each span can be assumed as simply supported, meaning $u\left(0\right)=u(L)=0$ where 0 and L refers to start and end of the girder.

## 3. Results and Discussion

### 3.1. Static Measurements with BOTDA

#### 3.1.1. Brillouin Frequency Shift

**Figure 3:**
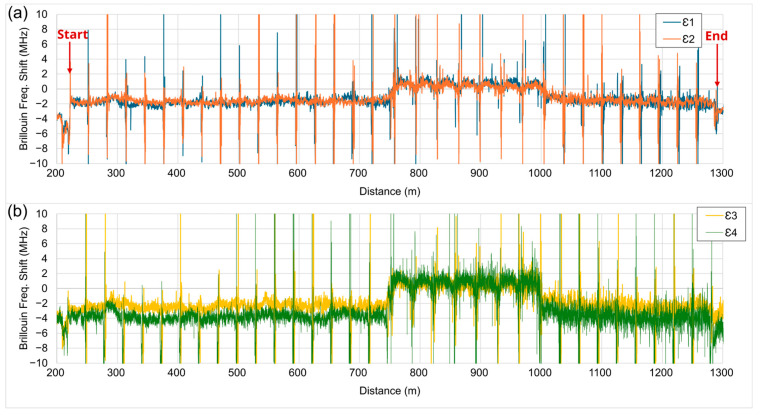
Brillouin frequency shift of four fibers between 7 A.M. 24 October and 2 P.M. 26 October, for (**a**) fibers on top half and (**b**) fibers on bottom half of box girder.

The initial reference data for the Brillouin frequency spectrum were taken on 24 October 2021 at 7 A.M. Taiwan time. Figure 3 shows an example of Brillouin frequency shift for four fibers on 26 October 2021 at 2 P.M. (over two days from reference). In the figure, large ‘spikes’ can be seen at equal spacing about 30 m along the X-axis, which corresponds to the expansion joints between the bridge girders. The expansion-joint strains were much higher than beam strains because the beam displacements were exerted over a short-length gap. For the girder displacement calculations, the expansion joint strains were omitted because they were the result of the relative motion of bridge spans rather than individual deformations. Measurement data from the beam fiber segments show good signal fidelity which confirms the good coupling of the fibers to the concrete. The Brillouin frequency shift shown in Figure 3 is raw data from the instrument, and they are sensitive to environmental noise such as wind, vibration, and temperature fluctuations. However, the integrations in displacement calculation Equations (4) and (5) have a low-pass filter effect which diminishes the high-frequency noise in the final displacement results.

#### 3.1.2. Bridge Displacement Results

**Figure 4:**
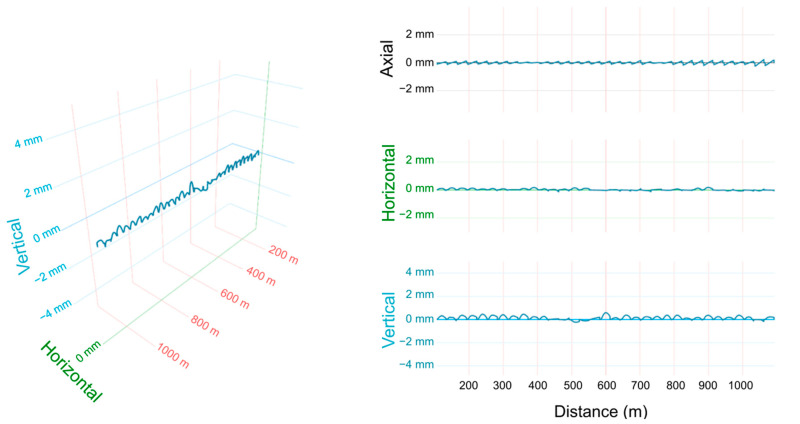
The deformation of the bridge structure in the entire monitoring interval. (**left**) Horizontal and vertical bending deformations and (**right**) each component of displacement.

The displacement of the bridge structure can be analyzed as the total deformation or separated into axial, horizontal, and vertical components. The overall picture of the bridge displacement at 1:10 A.M. on 26 October 2021 is shown in Figure 4. The displacement was around ±0.5 mm, regardless of axial (north–south), horizontal (east–west), or vertical (up-and-down) direction. The three-dimensional plot on the left of Figure 4 shows that there was no obvious change in the alignment of the bridge as a whole. The periodic displacement variations are the result of the almost independent deformation of each bridge girder since it is a common design in civil engineering to decouple different sections of a bridge to accommodate thermal expansion or contraction and to release bending moments. Each period (~30 m) corresponds to the length (free span) of the bridge girder.

**Figure 5:**
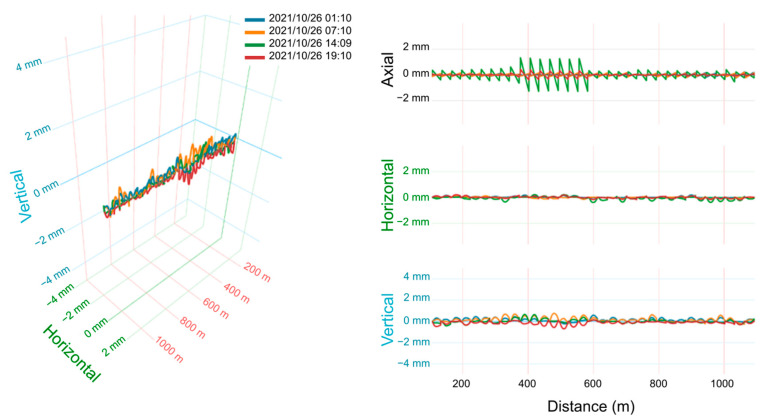
The deformation of the bridge structure in the entire monitoring interval.

Figure 5 shows the bridge deformation at different times of day from early morning to evening. The maximum displacement variation is within ±1 mm except for the high axial-displacement anomaly at 14:09 around 400 m to 600 m. This anomaly was deemed to be the result of temperature influence on that section. The vertical displacement component is consistently bigger than the horizontal component, which is reasonable considering that most loads from gravity and trains are in the vertical direction.

**Figure 6:**
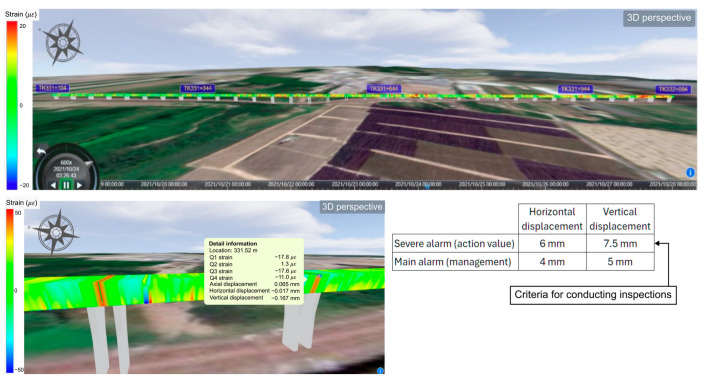
(**above**) An example display of the entire monitoring section by the SCADA system. (**below**) A display of a section of interest and the warning values for safety management and inspection implementation standards.

The entire data analysis process from measurement, strain conversion, and temperature compensation to displacement calculation was integrated into a Supervisory Control and Data Acquisition (SCADA) system which was specifically developed to interoperate with the DFOS system. This integration of sensing hardware and analysis software enabled real-time monitoring, data interpretation and automatic warning in the event that parameters exceeded safety standards. Figure 6 shows an example display of the bridge structure and detailed information of a section of particular interest from the SCADA system. The amount of measured strain is shown in color tones, and the detailed information shows the axial strain, axial displacement, horizontal displacement, and vertical displacement of each quadrant at a specific location. The standards for safety management were set based on the design and maximum allowable value; for example, one-third was the warning value and two-thirds was the inspection threshold. The specific values were 4 mm for horizontal and 5 mm for vertical displacements, respectively. As can be seen from Figure 5, the result of the deformation of the entire monitoring section was far below the warning value.

#### 3.1.3. Long-Term Monitoring Results

**Figure 7:**
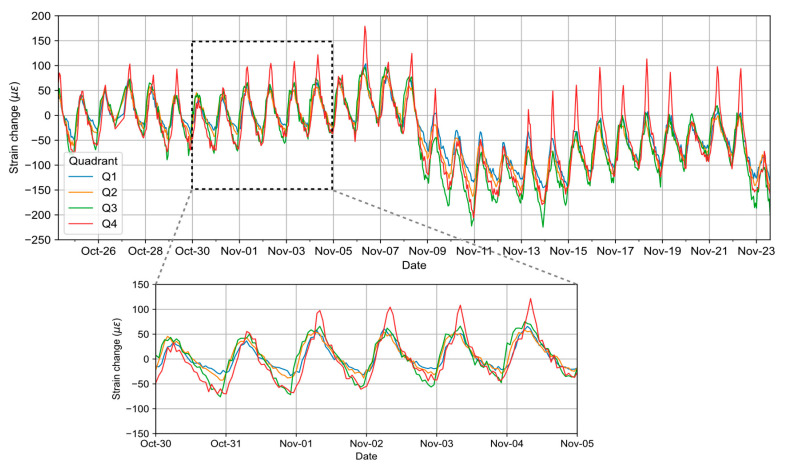
Strain changes over one-month interval (4-quadrant comparison at 700 m). Each tick on X-axis corresponds to start of day (00:00).

Structural health monitoring requires the recording and analysis of time-series data over the lifetime of the structure to identify trends and periodic patterns. Departure from the past trend pattern could indicate the deterioration of the structure or changing environmental factors, which may require detailed inspections and/or pre-emptive action depending on the extent of deviations. Strain monitoring is highly beneficial in this regard since a sudden increase in strain magnitude implies the structure is under undue stress and deformation. As an example of long-term monitoring, Figure 7 shows a one-month time series of fiber strains in four quadrants Q1 to Q4 (which corresponds to ε1 to ε4 in Figure 2) at a distance of 700 m. It can be observed that each day measures a variation of about ±50 µε on all four quadrants, which can be attributed to the thermal expansion of the bridge.

#### 3.1.4. Confirmation of Safety of Bridge Structure After an Earthquake

Earthquakes of Richter scale 6.4 and 6.8 occurred on 17 September 2022 and 18 September 2022 in eastern Taiwan. The local seismic intensity observed near the measurement region of the bridge was 5 and 3, respectively, on Taiwan’s 7-level earthquake scale. Immediately after the seismic event, damage confirmation called for the need to analyze and promptly confirm that there was no abnormality in the structure.

The structural integrity of the bridge could be quickly checked by comparing strain measurement data before and after each earthquake. Table 3 shows the timestamps of the earthquake events and the two measurement times used in each analysis. The bridge is vulnerable to bending from horizontal earthquake loads; hence, it is especially important to check the bending strains which are the strain differentials on opposite sides of the cross section in Figure 2. In addition, the width of the gaps between bridge girders needs to be checked to ensure there is no severe misalignment between individual girders. This can be verified from the magnitude of the fiber strains on the expansion joints before and after the earthquakes.

**Figure 8:**
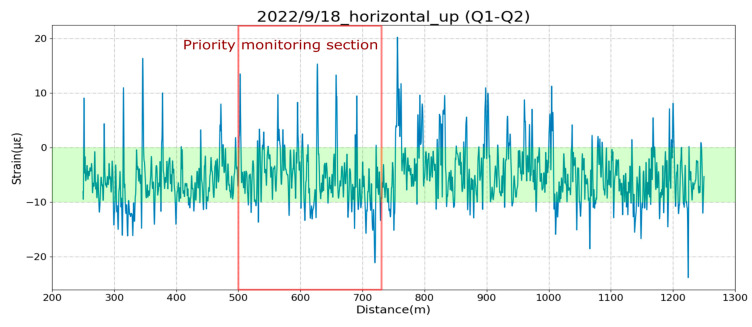
The horizontal bending strain in the upper part of the bridge girder after the earthquake on 18 September 2022. The green shaded region shows the normal range of strains before the earthquake.

**Figure 9:**
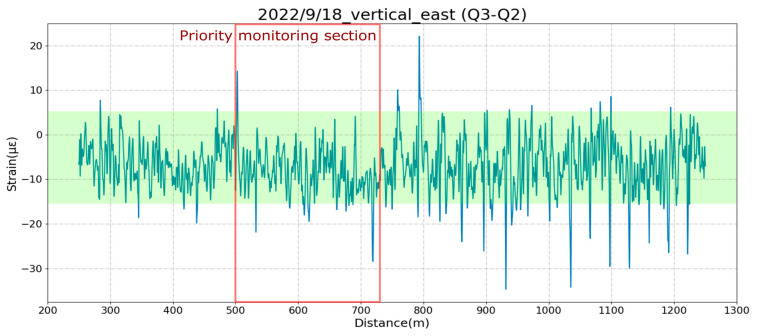
The vertical bending strain on the left side of the bridge girder after the earthquake on 18 September 2022. The green shaded region shows the normal range of strains before the earthquake.

Figure 8 shows the change in the bending strain of the upper half of the bridge (ɛ1–ɛ2) after the September 18 earthquake. The measured strains were less than 20 µɛ, which was much smaller than the 4000 με that corresponded to the warning value of 4 mm of displacement. Similarly, Figure 9 shows that the vertical-strain change was less than or equal to 20 με. The maximum strain of the connection joints was ±30 με, which was much less than the warning control value of −16,000~8000 με, corresponding to −16~8 mm displacement. In this way, it was quickly confirmed by the distributed optical fiber sensing system that safety was ensured after an earthquake.

### 3.2. Dynamic Measurements with DAS

**Figure 10:**
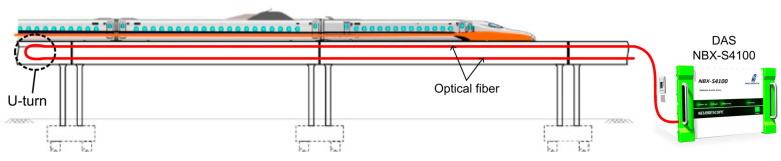
Conceptual diagram of dynamic measurement; fiber is U-turned at end of monitoring section.

**Figure 11:**
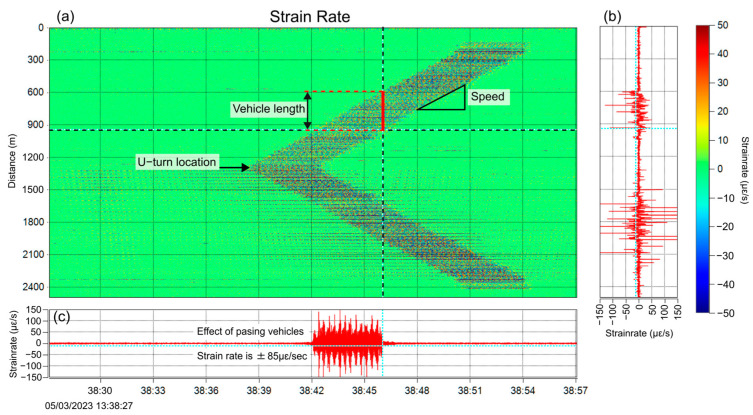
(**a**) The dynamic signal (strain rate) due to a passing train, measured by the DAS system, (**b**) the time snapshot along the vertical dotted line, (**c**) the time trace along the horizontal dotted line.

Using two of the optical fibers implemented for structural monitoring, dynamic measurements of the bridge behavior during train travel were performed. The system used was NBX S-4100 from Neubrex (Japan) with acquisition settings described in Table 2. Figure 10 shows a conceptual diagram of the measurement. The DAS interrogator measures the strain rate (rate of change in strain) and the result is shown as a color plot in Figure 11. The vertical axis is the fiber distance and the horizontal axis is time, and it can be clearly seen the train first passed the U-turn point and traveled along the fiber. The overall signal in the plot is shaped like an arrowhead (<) with opposite slopes at the top and bottom halves. This is because the optical fiber cable is folded back around the middle at 1300 m distance. The vertical width of the dark part indicates the length, and the angle represents the speed of the train. The inspection of the figure reveals that the vibration effect of the train load on the structure is approximately ±85 με/s.

**Figure 12:**
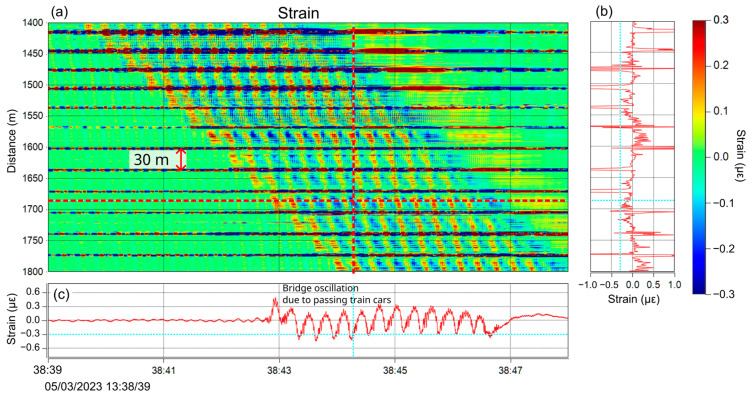
(**a**) The dynamic strain of the bridge due to a passing train, (**b**) the time snapshot along the vertical dotted line, (**c**) 13 peaks from the bogies of 12 train cars can be seen in the time trace.

In order to see the load effect of the passing train on the bridge, the strain rate was converted to strain by time integration, and the zoomed-in result is shown in Figure 12. The horizontal stripes on the plot have equal intervals of approximately 30 m, which are judged to be the position of the bridge’s expansion joints. Both ends of the bridge girder were supported by pillars and hence deformation was small near the pillars and larger at the middle. The time trace at the bottom shows 13 oscillation peaks which originated from the bogies of 12 train cars. In this way, the characteristics of the structure were clearly captured by DAS measurement during the passage of a high-speed train. This shows the possibility of clarifying the structural properties and dynamic response in detail using running trains and suggests that more advanced health monitoring is possible when combined with static monitoring.

## 4. Conclusions

The field trial data from static and dynamic DFOS measurements of a railway bridge structure in Taiwan were reported. The static measurements of strains were used to calculate bridge deformation in few-minute intervals, and safety confirmation after earthquakes was achieved from the rapid-measurement data. Dynamic monitoring with DAS revealed the vibration response of the bridge to the train bogies. The definitive results of this one-year-long field trial include bridge deformations, strain evolution history, and damage confirmation after a natural disaster. It can be expected that the applications of DFOS for structural health monitoring will continue to expand as more systems are deployed and evaluated in field conditions.

## Figures and Tables

**Table 1:** Acquisition parameters of Brillouin measurement (NBX-6065).

Total Distance	4 km (1 km × 4)
Spatial resolution	20 cm
Sampling interval	5 cm
Time interval	≈1.5 min
Strain accuracy (σ)	15 με
Temperature accuracy	0.75 °C
Measurement mode	PPP-BOTDA

**Table 2:** Acquisition parameters of DAS measurement (NBX-S4100).

Spatial resolution	1 m
Gauge length	1 m
Spatial sampling interval	1 m
Time sampling interval	0.001 s
Interrogation rate	1000 samples/s

**Table 3:** Measurement times for safety confirmation after earthquake.

Date of Earthquake	17 September 2022	18 September 2022
Richter scale	6.4	6.8
Time of earthquake	21:41	14:44
Pre-earthquake measurement time	21:00	14:00
Post-earthquake measurement time	22:00	15:00

## Data Availability

Data will be available on request to the corresponding author.
